# Reliability of E-Tests and the Phoenix Automated Method in Assessing Susceptibility to IV Fosfomycin—Comparative Studies Relative to the Reference Method

**DOI:** 10.3390/pathogens12050700

**Published:** 2023-05-12

**Authors:** Beata Kowalska-Krochmal, Beata Mączyńska, Danuta Smutnicka, Anna Secewicz, Grzegorz Krochmal, Klaudyna Laufer, Ruth Dudek-Wicher

**Affiliations:** 1 Department of Pharmaceutical Microbiology and Parasitology, Faculty of Pharmacy, Medical University of Silesian Piasts in Wroclaw, 50-556 Wroclaw, Poland; beata.maczynska@umw.edu.pl (B.M.); danuta.ruranska-smutnicka@umed.wroc.pl (D.S.); anna.secewicz@umed.wroc.pl (A.S.); grzegorz.krochmal@gmail.com (G.K.); r.dudek.wicher@gmail.com (R.D.-W.); 2 Laboratory Diagnostics Department, Jan Mikulicz-Radecki University Teaching Hospital, 50-556 Wroclaw, Poland; klaudyna.laufer@gmail.com

**Keywords:** IV fosfomycin susceptibility testing, reference agar dilution method, E-test, Phoenix BD system

## Abstract

The agar dilution method (ADM) recommended for IV fosfomycin (IV FOS) is complex and labor-intensive. Keeping in mind the reality of everyday laboratory work, we have evaluated the agreement of IV FOS susceptibility results obtained using the E-test and the Phoenix system with the results obtained using the ADM. Materials and methods: The tests were performed on 860 strains. To evaluate susceptibility to IV FOS, BioMerieux E-tests (bioMerieux, Warsaw, Poland), BD Phoenix panels (BD Phoenix, Sparks, MD, USA), and the ADM were used. Clinical interpretation was performed in accordance with *EUCAST Guidance* (v12.0, 2021). The significance of the E-test and the Phoenix was analyzed in relation to the ADM by defining categorical agreement (CA), major error (ME), and very major error (VME). Essential agreement (EA) has also been defined for the E-test. A method was considered reliable, in accordance with ISO 20776-2:2007, when CA and EA were above 89.9% and VME was <3%. Results: A categorical agreement of >98.9% was demonstrated between the E-test and the ADM for overall strains and for *Echerichia coli*, ESBL-producing *Enterobacterales*, and *Staphylococcus aureus*, while between the Phoenix and the ADM, a CA of >98.9% was shown only for *Escherichia coli*, *Staphylococcus aureus*, and *Proteus* spp. A very major error rate of <3% was obtained only for *Staphylococcus aureus* and MBL-producing *Pseudomonas* evaluated by both the E-test and the Phoenix. An essential agreement of >98.9% between the E-test and the ADM has not been demonstrated for any of the tested groups of strains. The Phoenix yielded more VMEs than the E-test (50 and 46, respectively). The highest VME rate was demonstrated using the Phoenix method for *Enterobacter* spp. (53.83%). Conclusions: Both the E-test and the Phoenix have turned out to be reliable in assessing IV FOS susceptibility only for *Staphylococcus aureus* (CA > 89.9% and VME < 3%). For the remaining tested groups of strains and genera, the simultaneous high CA rate and low VME rate required by ISO were not achieved. Both methods fared particularly badly in detecting strains resistant to IV.

## 1. Introduction

Effective treatment of bacterial infections requires the satisfaction of numerous criteria. The administered antibiotic must penetrate to the site of infection, reach a therapeutic concentration there, and be directed against a susceptible bacterial pathogen. It is obviously not easy, especially for seriously ill patients with unpredictable pharmacokinetic parameters affecting the fate of the drug in the human body. The problem is exacerbated by the resistance of bacteria to antibiotics, which is both on the rise and difficult to predict without microbiological tests. It is impossible to determine the cause of infection and the sensitivity profile of the pathogen only on the basis of the clinical picture, even if the assessment of inflammatory parameters has been taken into account. Therefore, therapeutic decisions regarding patients with infections must be supported by the results of microbiological testing. However, for such results to be clinically useful, microbiological diagnostics should be carried out using diagnostically valuable clinical samples and reliable methods, including those used to evaluate the sensitivity of bacteria to antibiotics. The performance of an antibiogram used to be much easier for laboratories doing routine microbiological testing. For many years, methods such as the disc diffusion method or the quantitative method using strips impregnated with antibiotic concentration gradients have been recognized as standards in the study of most types of bacteria and antibiotics. The above also applies to automated systems based on the broth microdilution method, such as the BD Phoenix (Sparks, MD, USA) or BioMerieux VITEK (bioMerieux, Warsaw, Poland), which can be conveniently used in routine diagnostics. Although automated tests do not allow for the ascertainment of the actual MIC, they have been regarded as reliable in determining drug susceptibility categories. Today, however, the evaluation of bacterial susceptibility to antibiotics has definitely become more difficult. Constantly updated EUCAST and CLSI recommendations limit the possibilities of using individual methods depending on the antibiotics and pathogens tested. The disc diffusion method should not be used to evaluate susceptibility to tigecycline and fosfomycin (except for *E. coli*), to glycopeptides and lipoglycopeptides in *Staphylococcus* spp., to colistin, to all antibiotics for *Neisseria* spp. and anaerobes, to dalbavancin and oritavancin for *Viridans*, and to the A, B, C, and G groups of *Streptococcus*. Antibiotic-impregnated strips are currently considered unreliable in determining the sensitivity of Gram-negative bacilli to colistin and cefiderocol and of *Staphylococcus* spp. to glycopeptides and lipoglycopeptides [1,2,3]. The gradient method affords lower colistin MIC values than the reference broth microdilution method. The reasons are the limited capability of polymyxin for diffusion from the strip into the agar due to its particle size as well as a possible interaction with the plastic material used to make the strip [4,5]. The use of this method for the evaluation of *Staphylococcus* spp. susceptibility to vancomycin is associated with higher MIC values by 0.5–1 of a twofold dilution [6]. The broth microdilution method, on the other hand, is considered inappropriate for fosfomycin and mecillinam [1,2,3]. The agar dilution method has been recognized by CLSI and EUCAST as the only reliable method for the determination of susceptibility to fosfomycin, with the exception of *E. coli*, for which the disc diffusion method has also been approved [1,2]. The above decisions were based on the research by Fuchs et al. [7], who found that the broth dilution method did not produce repeatable MIC values in subsequent determinations and led to large discrepancies in the MIC readings themselves. A high dependence of fosfomycin MIC has been demonstrated on the variable bacterial inoculum described in the case of the broth microdilution method, falling within a wide range of 1.6 × 10^5^ to 1.2 × 10^6^ CFU/mL [7,8]. Currently, in accordance with the results of many studies, it is considered that, compared to the agar dilution method, other methods all too often produce false results in the determination of susceptibility to fosfomycin. However, it should also be emphasized that, as pointed out by Ballestero-Tellez M. et al. [9], there are practically no studies on the correlation between the fosfomycin MIC value determined in vitro by various methods, including the reference method, and clinical efficacy.

Owing to its pharmacokinetic and pharmacodynamic properties, fosfomycin has been increasingly considered when making therapeutic decisions [10,11,12,13,14,15]. Fosfomycin is available in oral form (trometamol) and intravenous form (disodium salt). Both forms achieve high urine concentrations, significantly exceeding 128 mg/L. However, fosfomycin serum concentrations vary significantly depending on the form of the drug applied. It has been shown that 2.5 h after oral administration of three grams of fosfomycin trometamol, the serum concentration C_max_ was 21.8 ± 4.8 mg/L, whereas after administration of the same dose of fosfomycin intravenous formulation, the serum concentration was 370.6 ± 92 mg/L [16]. The fraction recovered in urine after oral administration is 32–43% and after intravenous administration is more than 85% [17].

Among the reasons for intravenous fosfomycin’s popularity are its bactericidality, broad spectrum of action, penetration into most organs and tissues, including the CNS, as well as potential activity against multi-resistant bacteria, such as ESBL-producing or carbapenemase-producing bacilli or methicillin-resistant staphylococci [10,11,12,13,14]. Good penetration into many organs and the possibility of application in various infections are the properties of intravenous fosfomycin, whose significance has been growing in recent years, especially in combating multi-resistant bacteria. IV fosfomycin also has the ability to penetrate into macrophages and osteoblasts and further kill them intracellularly [15,16,17,18,19]. Its possible penetration through bacterial biofilm is also mentioned [18,19]. It is approved for use in both adults and children (regardless of age), primarily in severe infections not susceptible to other antibiotics. Fosfomycin inhibits cell wall synthesis by blocking UDP-N-Acetylglucosamine Enolpyruvyl Transferase (MurA). This mechanism is different from the mechanism of action of other antibiotics on the cell wall; hence, no cross-resistance between fosfomycin and other antibacterial drugs is observed. Therefore, the evaluation of bacterial susceptibility to IV fosfomycin has become a necessity due to the increasing share of Gram-negative bacilli resistant to most antibiotics in infections as well as the growing frequency of infections of mixed etiology (involving Gram-positive and Gram-negative bacteria). Unfortunately, the reference method (agar dilution) dedicated to fosfomycin is the most difficult among the available drug susceptibility testing methods and is practically impossible to use in routine diagnostics. Instead, laboratories test fosfomycin susceptibility using the gradient diffusion method (e.g., E-test, M.I.C.E.) or automated systems (although they are not recommended). Taking into account the reality of routine drug susceptibility testing as well as various reports on the value of individual methods of determining sensitivity to fosfomycin, our research aimed to assess the consistency of results obtained using the E-test method and the automated BD Phoenix system with the results obtained using the reference agar dilution method (ADM).

## 2. Materials and Methods

A total of 860 bacterial strains were tested, including bacteria from the *Enterobacterales* order (*n* = 546), including *Klebsiella* (*n* = 250), *E. coli* (*n* = 181), *Enterobacter* spp. (*n* = 47), *Proteus* spp. (*n* = 41), other *Enterobacterales* spp. (*n* = 27), and strains of *Pseudomonas* (*n* = 153) and *Staphylococcus* (*n* = 161), including *S. aureus* (*n* = 104) and coagulase-negative *Staphylococcus* spp. (CNS) (*n* = 57). The tested strains were isolated mainly from urinary tract infections (*n* = 224), wounds (*n* = 218), blood (*n* = 198), the lower respiratory tract (*n* = 94), and intraabdominal infections (*n* = 41). Apart from the above, they were isolated from the eye, ear, uterine cervix, or amniotic fluid.

Among the studied Gram-negative bacilli were: ESBL-producing *Enterobacterales* (*n* = 139 strains), carbapenemase-producing *Enterobacterales* (CPE) (*n* = 60), and metallo-beta-lactamase (MBL)-producing *P. aeruginosa* (*n* = 21). Among CPE strains, 50 produced NDM-1 enzymes, 6 produced VIM enzymes, and 4 produced OXA-48 enzymes.

### 2.1. Agar Dilution Method—The Reference Method

The study was performed in accordance with EUCAST Definitive Document E. Def. 3.1 2000 [20].

**Figure 1:**
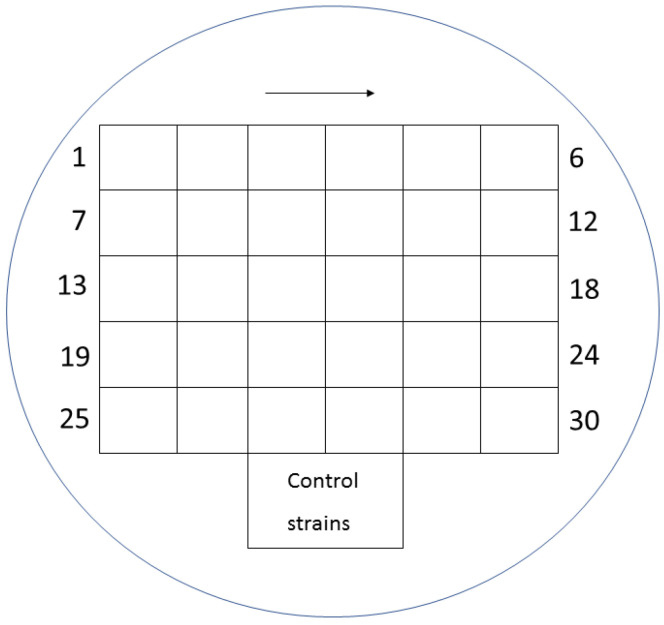
Distribution of individual suspensions of various bacteria on a medium with fosfomycin.

**Figure 2:**
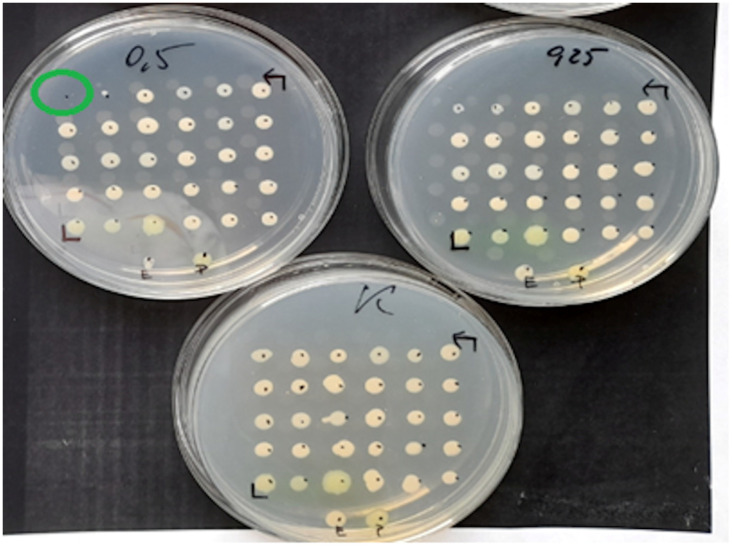
Example MIC reading using the ADM on a medium inoculated with 30 test strains and 2 reference strains of *E. coli* (ATCC 25922) and *P. aeruginosa* (ATCC 27853). The figure shows the growth control medium for the tested strains and the medium with fosfomycin at concentrations of 0.25 mg/L and 0.5 mg/L. One of the strains has a MIC of 0.5 mg/L.

**Figure 3:**
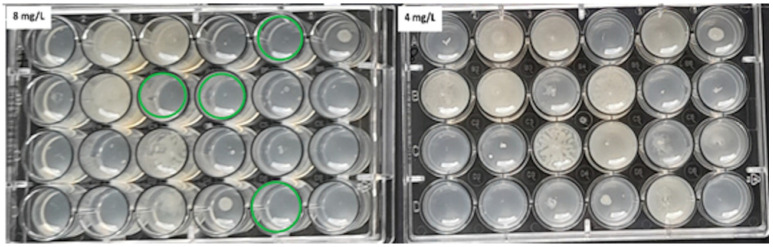
The ADM method for *Proteus* spp. The green circles indicate MIC values of 8 mg/L for some of the tested strains.

Mueller-Hinton agar (MHA) (BioMerieux, Warsaw, Poland) enriched with glucose-6-phosphate at a concentration of 25 mg/L (Sigma-Aldrich, Saint Louis, MO, USA) was used for the evaluation. The working solutions of fosfomycin were prepared according to the EUCAST procedure [20] and were added to the previously prepared MHA so as to finally obtain concentrations of the antibiotic in the range of 0.25–512 mg/L. MHA media supplemented with glucose-6-phosphate and successive concentrations of the antibiotic were poured into petri dishes. Each of the plates allowed for the evaluation of 30 bacterial strains. Two µL of the 10^7^ CFU/mL suspension obtained by 10-fold dilution of the 0.5 McFarland suspension was applied to the plates. In this way, the required inoculum of 10^4^ CFU per spot was achieved. Taking into account the swarming growth of these rods, for *Proteus* spp., 24-well plates were used, modeled after the commercial method [21]. Each isolate was inoculated in technical triplicate on plates with fosfomycin. Where the results were inconsistent, the MIC value that occurred in at least two replicates was selected. Assays were evaluated after 18 ± 2 h of incubation at 35 ± 1 °C. The value at which growth was invisible to the naked eye was regarded as the MIC. A growth of 1–2 colonies or faint turbidity due to bacterial inoculum was ignored. For each strain, a growth control on MHA without an antibiotic was performed. The arrangement of individual suspensions of the tested bacteria on the medium with the antibiotic is shown in Figure 1, and examples of MIC readings are shown in Figure 2 and Figure 3 (for *Proteus* spp.).

### 2.2. Gradient Diffusion Method

The study used BioMerieux E-tests containing fosfomycin in the concentration range of 0.064–1024 mg/L, BioMerieux MHA media, and a suspension of bacteria with a density of 0.5 McFarland standard. After about 15 min, after the suspension had dried, E-tests with fosfomycin were applied. The tests were incubated at 35 ± 2 °C for 16–20 h and, in the case of *Staphylococcus* spp., for 20–24 h. MIC readings were performed according to the manufacturer’s instructions and depended on the tested strain type [22].

For *Enterobacterales*, the MIC was read at 80% growth inhibition (the first point of significant inhibition by the naked eye). Turbidity, macrocolonies, and microcolonies were not taken into account during the reading, except when the colonies filled the entire ellipse; then the maximum MIC for this method was determined to be ≥1024 mg/L [22]. The same assessment criteria were adopted for *Pseudomonas aeruginosa* despite the lack of relevant guidance in the manufacturer’s instructions.

**Figure 4:**
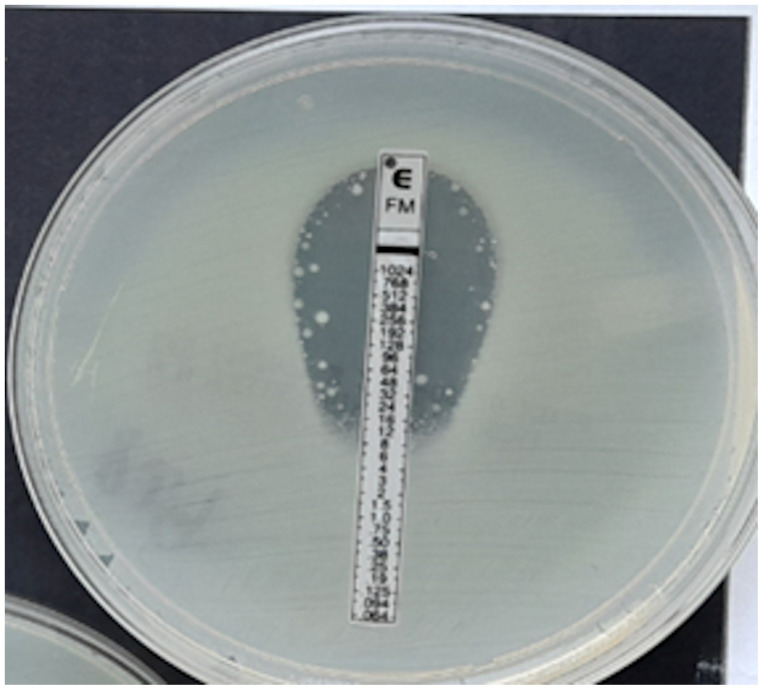
ESBL-producing *Enterobacter cloacae* strain, MIC = 12 mg/L.

**Figure 5:**
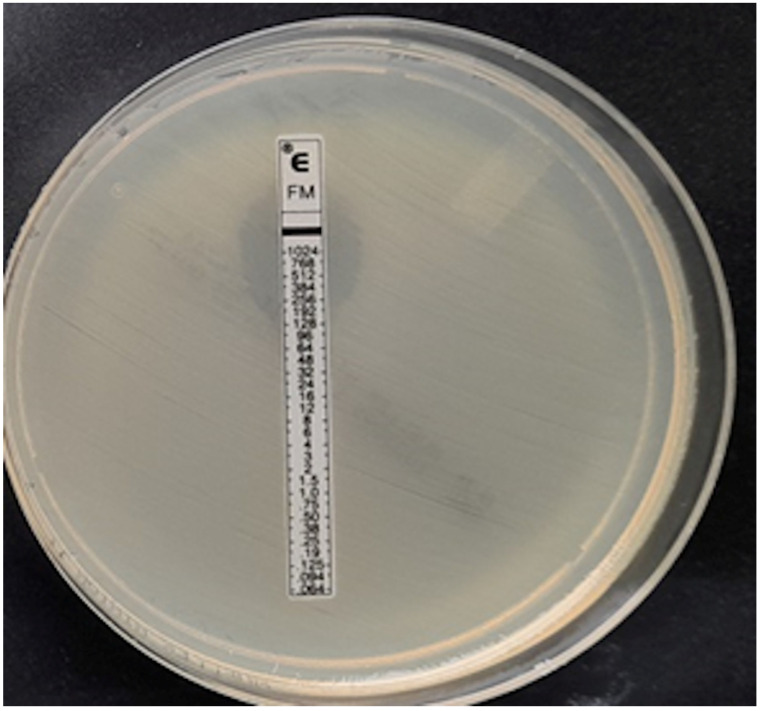
*Klebsiella pneumoniae*, MIC > 1024 mg/L.

**Figure 6:**
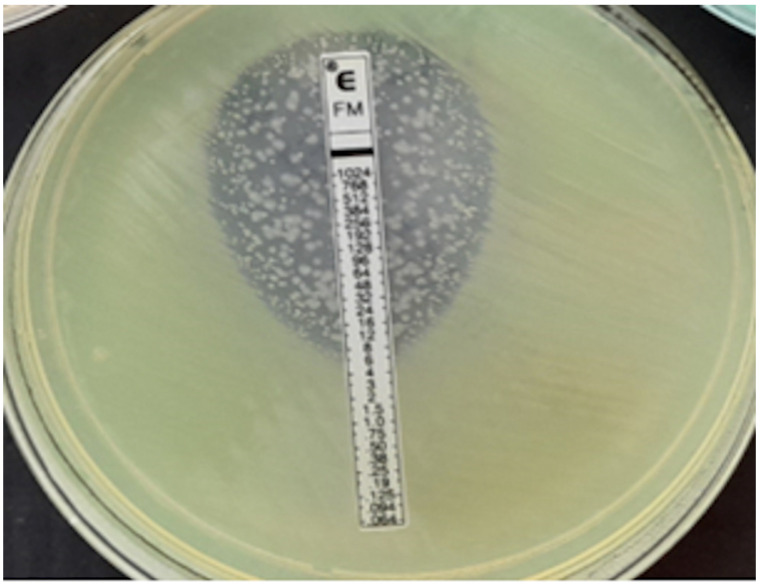
*P. aeruginosa*, MIC = 1024 mg/L.

**Figure 7:**
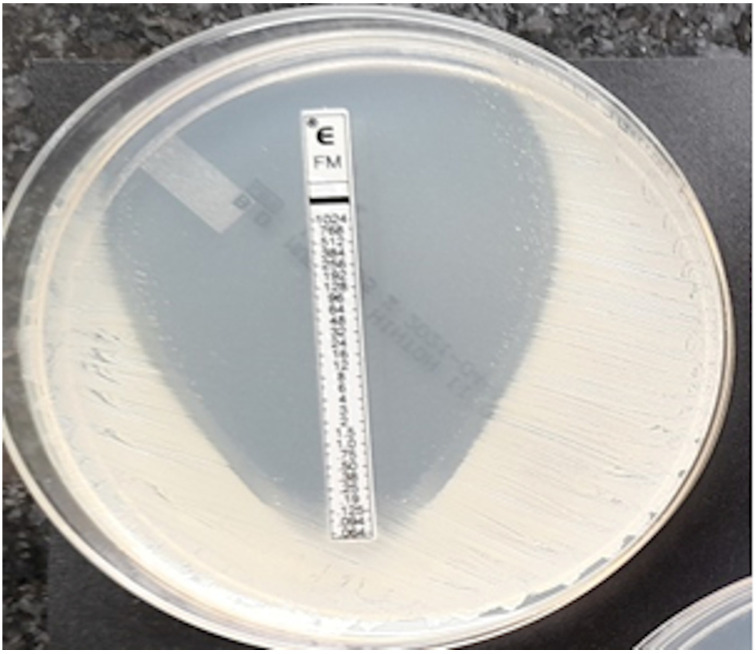
Coagulase-negative strain of *Staphylococcus*, MIC = 0.125 mg/L.

**Figure 8:**
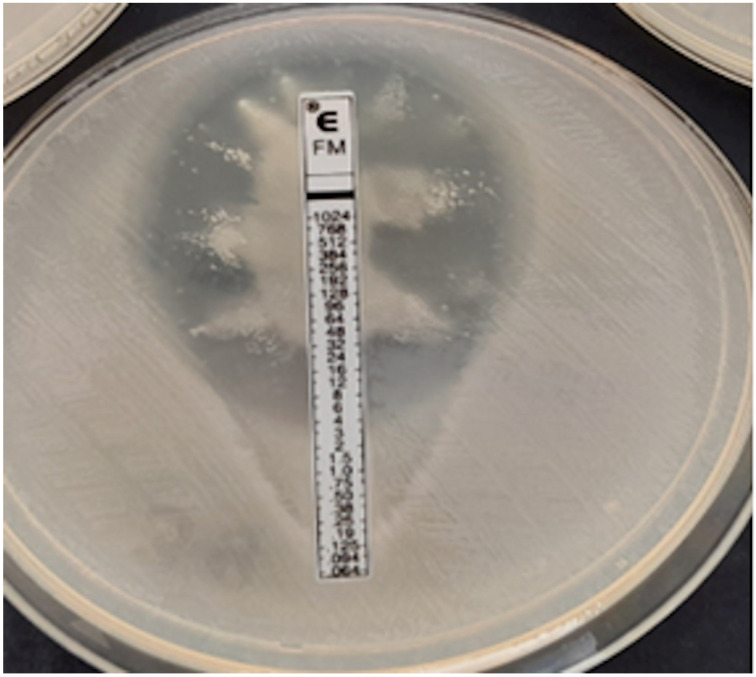
*Proteus* spp. swarming growth, no MIC readable.

In the case of *Staphylococcus* spp., the MIC was read at the point of complete inhibition of bacterial growth. Turbidity, macrocolonies, or microcolonies within 3 mm of the strip were treated as growth [22]. Examples of MIC determination using fosfomycin E-tests are shown in Figure 4, Figure 5, Figure 6, Figure 7 and Figure 8.

The MIC values of the E-test have been scaled to the ADM to allow for comparison. The MIC values of the ADM are represented by a geometric sequence (*first term* = 0.25, *common ratio* = 2). To allow for comparison, the MIC values of the E-test are scaled up to the closest ADM-MIC value (e.g., E-test = 3 becomes 4).

### 2.3. Evaluation of Fosfomycin Susceptibility Using BD Phoenix Panels

In the tests, we used the automated method in the Phoenix 100 system (Becton Dickinson, Sparks, MD, USA) based on microdilution in a liquid medium. For *Enterobacterales*, NMIC/ID-402 panels (Becton Dickinson, Sparks, MD, USA)were used to assess MIC values in the range of 16–64 mg/L, and for *P. aeruginosa*, NMIC/ID-502 panels were used to determine MIC values 16–128 mg/L. For *Staphylococcus* spp., PMIC/ID-90 panels were used, allowing for MIC determination in the range of 16–64 mg/L. For each method, the obtained MIC values were subjected to clinical interpretation (Table 1) in accordance with the criteria contained in the EUCAST v.12 2022 Guidance [23].

In order to assess the reliability of the E-test and Phoenix methods in determining IV fosfomycin susceptibility, the results obtained with these methods were compared with the results produced by the ADM.

The following values were determined:

- categorical agreement (CA)—agreement regarding clinical interpretation.
- major errors (ME) when the strain turns out to be sensitive using the ADM and resistant using the evaluated method.
- very major errors (VME) when the strain is determined as resistant in the ADM and susceptible in the method being evaluated.
- the ME and VME rates—calculated by dividing the number of errors by the total number of susceptible or resistant isolates, respectively, obtained by the reference method.
- differences in MIC values obtained by the E-test in relation to the ADM reference method. The occurrence of deviations of one, two, or more than two two-fold dilutions was analyzed; this analysis was performed only for the E-test vs. the ADM.
- essential agreement (EA)—agreement within plus or minus one two-fold dilution of the E-test with the reference method; this analysis was performed only for the E-test vs. the ADM.

In accordance with ISO 20776-2:2007, a method was considered reliable if the CA and EA were >89.9% and the VME was <3% compared to the reference method [24].

The statistical and visual analysis has been conducted in the Python programming language version 3.9 and the following libraries: Numpy, Pandas, SciPy, Matplotlib, and scikit-learn (https://www.python.org/).

## 3. Results

Table 2 presents the results of fosfomycin susceptibility testing, depending on the method used. Overall, the percentage of antibiotic susceptibility obtained by the E-test and the Phoenix was comparable to the results obtained using the ADM, with differences not exceeding 1%.

However, when the analysis was performed separately for individual bacterial groups, significantly more susceptible strains were found using the E-test and Phoenix methods compared to the ADM for *Enterobacterales*, including ESBL-producing and carbapenemase-producing *Klebsiella* spp. and *Enterobacter* spp. In the case of *Pseudomonas* spp., on the other hand, both the E-test and the Phoenix returned a lower percentage of susceptible strains than the ADM. A comparable percentage of susceptibility was found for *E. coli* and an identical one in all three methods only for *S. aureus.*

### 3.1. Assessment of Categorical Agreement (CA) and Error Rates for the E-Test and the Phoenix vs. the ADM

**Figure 9:**
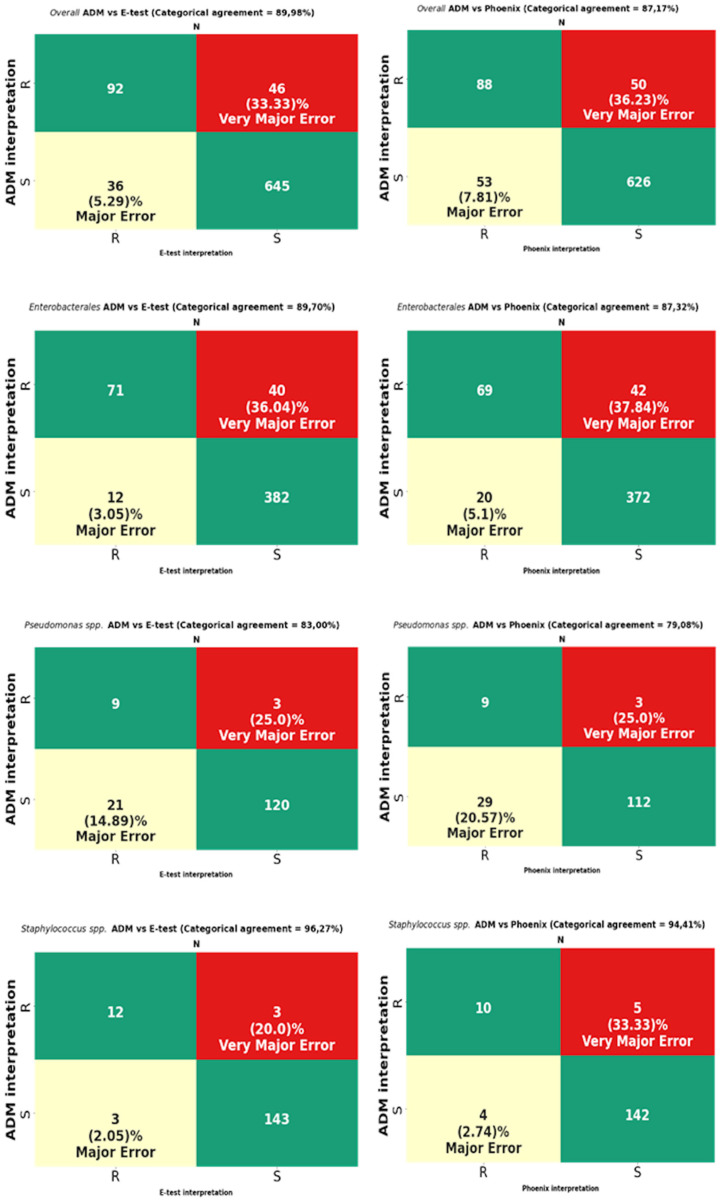
Categorical agreement and error rates of the ADM compared to those of the E-test and the Phoenix for fosfomycin for 819 overall strains: 505 *Enterobacterales* (except *Proteus* spp.), 153 *Pseudomonas* spp., and 161 *Staphylococcus* spp.

**Figure 10:**
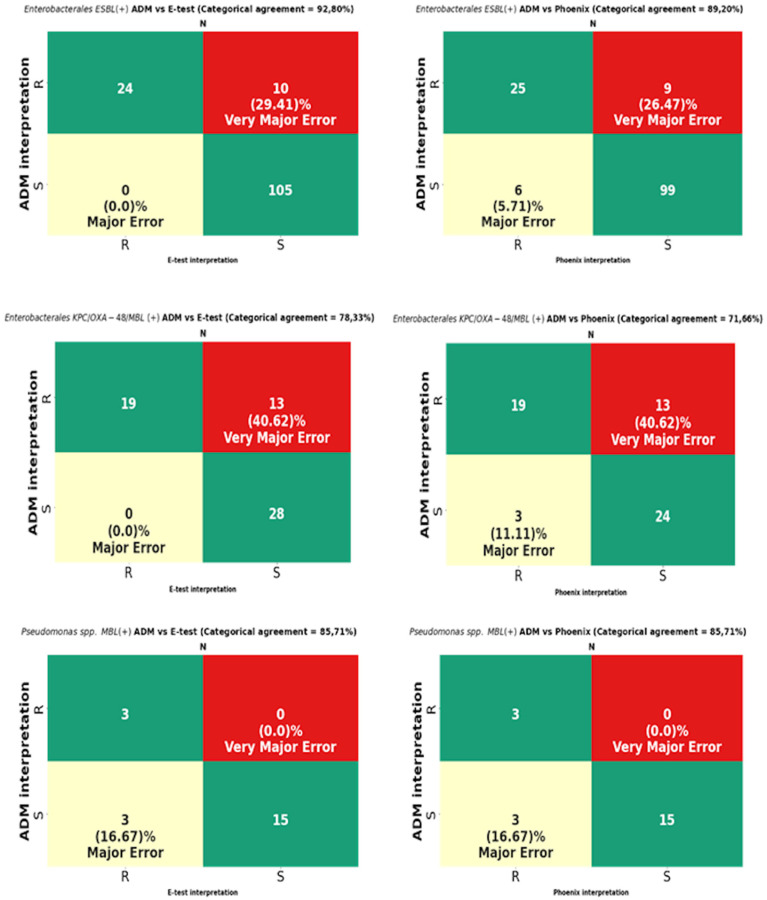
Categorical agreement and error rates of the ADM compared to those of the E-test and the Phoenix for fosfomycin for multidrug-resistant Gram-negative bacilli: 139 ESBL-producing *Enterobacterales*, 60 *Enterobacterales* producing at least one carbapenemase type (KPC/OXA-48/MBL), and 21 MBL-producing *Pseudomonas* spp.

The analysis was performed on 819 strains. Strains of the genus *Proteus*, for which a reliable reading of MIC by the E-test turned out to be impossible due to swarming growth, were excluded from the analysis (Figure 8). For *Proteus* spp., only a comparative analysis of the ADM vs. the Phoenix results was performed. The results of CA, MA, VME, and rate of errors are shown in Figure 9 and Table 3, and for multi-resistant Gram-negative bacilli in Figure 10.

MIC data for fosfomycin obtained using the E-test and the Phoenix enabled the obtaining of agreement regarding susceptibility category (CA) for all tested strains at the levels of 89.98% (737/819) and 87.17% (714/819), respectively (Figure 9). The CA of the E-test was higher for most of the tested groups of bacteria than the CA of the Phoenix, with the greatest differences in favor of the gradient diffusion method found in the case of testing the susceptibility of *P. aeruginosa* (by 4%) and CNS (by 5.26%). A CA that was comparable for both methods was found for *S. aureus* at 100% (Table 3) and MBL-producing *Pseudomonas* at 85.71% (Figure 10). The analysis of the CA for multi-resistant Gram-negative bacilli (Figure 10) has shown that in the carbapenemase-producing *Enterobacterales* group, the E-test and the Phoenix contributed to lower CA levels (78.33%-47/60 and 71.66%-43/60, respectively) and to a higher CA in the group of ESBL-producing bacilli (92.8%-129/139 and 89.2%-124/139, respectively), compared to the results for all *Enterobacterales* tested by the same methods (Figure 9).

The tests performed using the E-test and the Phoenix produced results riddled with VMEs.

The VME rates of the E-test were 36% (40/114) for *Enterobacterales* through 33.3% (46/138) for all tested bacteria, 20% (3/15) for *Staphylococcus* spp., and 0% for *S. aureus* and MBL-producing *Pseudomonas* (Figure 9, Table 3). A detailed analysis of genera within the *Enterobacterales* order has shown that VME% was even higher for *Klebsiella* spp. (39.53%-34/86), and the analysis within the *Staphylococcus* species has shown that all identified VMEs were obtained for the tested CNS (Table 3). The study of multi-resistant strains showed the highest VME rate of the E-test for carbapenemase-producing *Enterobacterales* (40.6%-13/32).

The VME rates of the Phoenix were 37.8% (42/114) for *Enterobacterales* through 36.2% (50/138) for all tested bacteria, and 25% (3/12) for *P. aeruginosa*. Among *Enterobacterales*, the highest VME rate was found for *Enterobacter* spp. (53.8%-7/13), and the lowest for *E. coli* (about 9%-1/11). Similar to the E-test, the VME rate was particularly high in the evaluation of fosfomycin susceptibility in carbapenemase-producing *Enterobacterales* (40.62%-13/32) (Figure 10). Additionally, no VMEs were found in the Phoenix during the evaluation of *S. aureus* and MBL-producing *Pseudomonas* strains.

In total, VMEs were found for 46 strains tested using the E-test method and for 50 strains evaluated using the automated Phoenix system.

### 3.2. Evaluation of Agreement of MIC Values Obtained Using the E-Test and the Reference ADM

**Figure 11:**
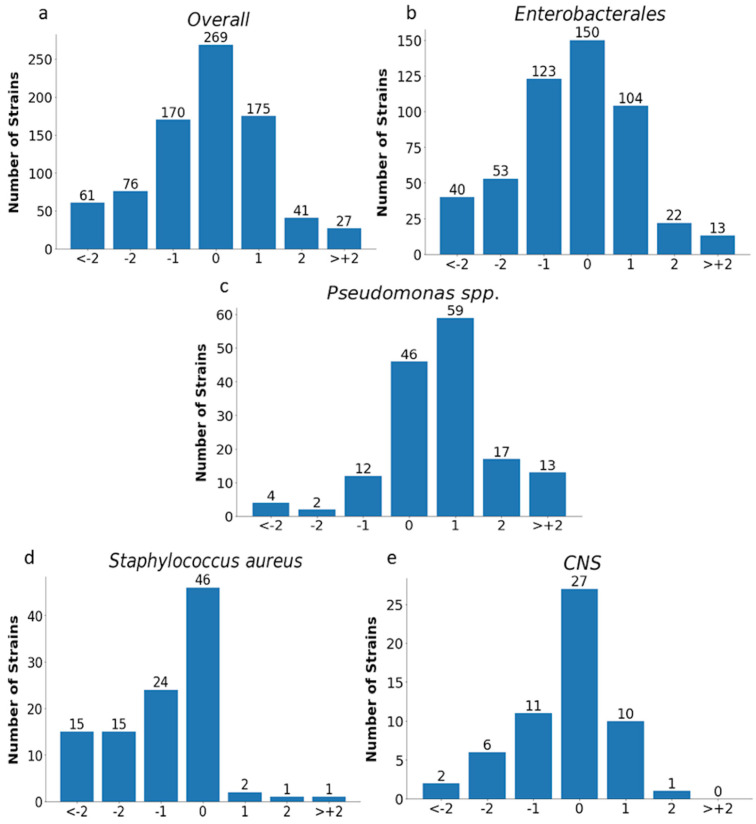
MIC deviations obtained by the E-test compared to the reference method. Number of results with one, two, or more than two two-fold dilutions for overall strains (*n* = 819) (**a**), *Enterobacterales* (*n* = 505) (**b**), *Pseudomonas* spp. (*n* = 153) (**c**), *Staphylococcus aureus* (*n* = 104) (**d**), and CNS (*n* = 57) (**e**); −1, −2, > −2, 1, 2, 3—the deviations of one, two, or more than two two-fold dilutions bellow or above the MIC in comparison to ADM method (0).

**Figure 12:**
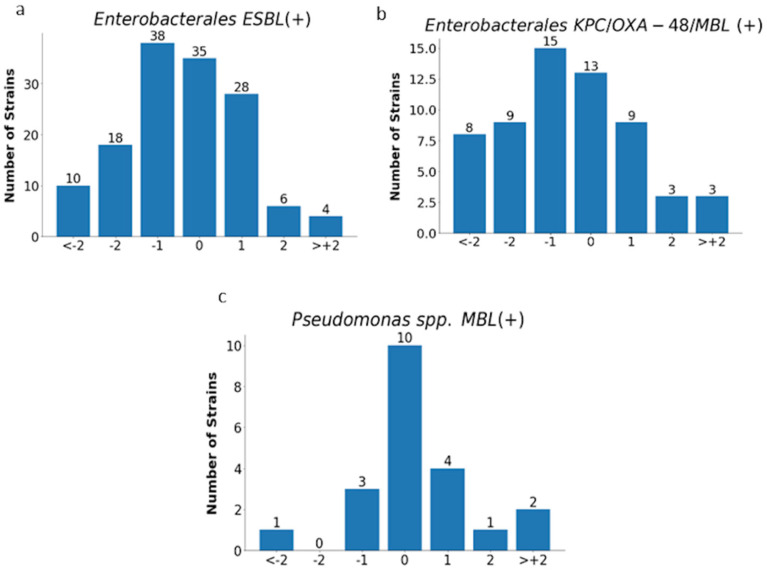
MIC deviations obtained by the E-test compared to the reference method. Number of results with one, two, or more than two two-fold dilutions for multidrug-resistant rods: ESBL-producing *Enterobacterales* (*n* = 139) (**a**), carbapenemase-producing *Enterobacterales* (*n* = 60) (**b**), and MBL-producing *Pseudomonas* spp. (*n* = 21) (**c**);−1, −2, > −2, 1, 2, 3—deviations of one, two, or more than two two-fold dilutions bellow or above the MIC in comparison with ADM method (0).

The analysis of MIC values obtained with the E-test was carried out for all tested strains (*n*= 819) excluding *Proteus* spp. and for individual groups of microorganisms, as shown in Figure 11 and Figure 12 and in Table 4.

Essential agreement (EA) in the E-test for all tested strains was 75% (614/819). Similar EA levels of 75% and 76% were found for *Enterobacterales* (377/505) and *P. aeruginosa* (117/153), respectively; slightly lower for *S. aureus* at 70% (72/104); the lowest for carbapenemase-producing *Enterobacterales* at 62% (37/60); and the highest for CNS at 84% (48/57) (Figure 11 and Figure 12 and Table 4). Differences of at least two two-fold dilutions were most often found for carbapenemase-producing *Enterobacterales* (38%-23/60), *Klebsiella* spp. (29%-73/250), and ESBL-producing strains (27%-38/139), and least often for MBL-producing *Pseudomonas* (19%-4/21) and CNS (16%-9/57) (Table 4).

Evaluation of EA and MIC differences for the Phoenix system has not been performed due to the limited range of MIC values that can be determined using this method and the much larger range evaluated by the reference method. In addition, extreme MIC values of <16 mg/L and >64 mg/L (NMIC/ID-502 panel) or >128 mg/L (NMIC/ID-408 panel) are practically incomparable to the actual MIC values in the ADM. Such an analysis would involve a large falsification of the EA. It can be justified by the examples of *E. coli* and *S. aureus*. Using the Phoenix, the lowest possible determinable MIC value is <16 mg/L, and such a value was presented by 91% (165/181) of *E. coli* and 97% (100/104) of *S. aureus* strains in this method, while 75% (136/181) of *E. coli* and 88% (92/104) of *S. aureus* in the ADM had a MIC value of ≤2 mg/L, so the difference to be recorded would be at least two two-fold dilutions of the antibiotic. Ultimately, it would yield a low but probably false percentage of EA.

## 4. Discussion

Intravenous fosfomycin (IV FOS) is an old antibiotic. However, owing to its very good pharmacokinetic properties and activity against the most resistant Gram-negative bacilli, this antibiotic is suggested as a potential therapeutic option in severe infections, next to the latest agents such as ceftazidime-avibactam, meropenem-vaborbactam, or cefiderocol. It is mentioned in combination therapy in infections with ESBL-producing strains as an alternative treatment regimen to carbapenems against carbapenemase-producing Gram-negative bacilli, colistin-resistant Gram-negative bacilli, difficult-to-treat *P. aeruginosa*, or infections with methicillin-resistant staphylococci [25,26,27,28,29,30]. High sensitivity to fosfomycin has been demonstrated in many studies, including our own published in 2022 [31]. At that time, nearly 78% of susceptible strains were found among *Enterobacterales* and nearly 91% in *Pseudomonas* spp. and *Staphylococcus* strains [31]. In addition, multi-resistant strains, such as MBL-producing *Pseudomonas* spp. or ESBL-producing bacilli, were highly sensitive to fosfomycin, nearly 86% and 76%, respectively. A lower percentage of susceptibility was shown in the group of *Klebsiella* spp. (66%) and in the carbapenemase-producing *Enterobacterales* (CPE) (47%). Most of these strains were also the research material in the currently presented study, this time focusing on the comparison of the E-test and Phoenix methods with the ADM in a reliable evaluation of susceptibility to IV fosfomycin. High sensitivity to fosfomycin may justify its use in combination therapy for severe infections. It is known, however, that the selection of antibiotics for therapy should be based on the results of microbiological tests. However, it becomes difficult in the case of fosfomycin because the performance of a sensitivity test for this drug using the reference method is quite a challenge for microbiologists. Therefore, physicians can rarely rely on fosfomycin susceptibility results obtained using the ADM. The above is undoubtedly an obstacle to making accurate therapeutic decisions. Therefore, the credibility of other methods that are simpler to perform, such as the gradient diffusion method or automated systems such as the BD Phoenix or BioMerieux VITEK, is currently being discussed. The performance criteria of the FDA and ISO 20776-2:2007 set high requirements and thresholds for categorical agreement (CA), essential agreement (EA), and very major errors (VME) for methods compared with the reference method [24,32]. For a method to be considered reliable, it should obtain a CA and EA of >89.9% and a VME of <3% (according to the ISO) or <2% (according to the FDA) compared to the reference method [22,30]. In our own research, the required level of CA was obtained with the E-test for the entire genus *Staphylococcus* (96.27%-155/161), including *S. aureus* (100%-104/104) and *E. coli* (96.68%-175/181), for ESBL-producing *Enterobacterales* (92.80%-129/139), and for overall tested strains (89.98%-737/819). Unfortunately, the E-test CA for *Klebsiella* spp. (Table 3), *Pseudomonas* spp. (Figure 9), CPE strains, and MBL-producing *Pseudomonas* strains (Figure 10) were lower than the required values by about 6%, 7%, 5%, 12%, and 4%, respectively. In our own research, using the E-test, we have not obtained an EA exceeding 89.9% for any of the groups of bacteria. Indeed, the obtained values were much lower and ranged from 62% (37/60) for CPE to 84% (48/57) for CNS. A VME rate of <3% was found only for *S. aureus* (0%) and MBL-producing *Pseudomonas* spp. In total, 46 VMEs were found in the group of 819 tested strains, including 40 in *Enterobacterales*, three in *P. aeruginosa*, and three in CNS. These numbers translated into a very high VME rate, amounting to 36.04% (40/114), 25% (3/12), and 21% (3/14), respectively, for the above-mentioned bacteria.

Other authors have also studied the CA between the E-test and the ADM. Results similar to ours were achieved by Van den Bijllaard et al. in their studies on *E. coli* and *Klebsiella* spp., who reported a CA of 99% and 84.8%, respectively [33]. These authors also showed a high VME rate (although lower than in our study), amounting to about 23% for both types of bacteria [33].

Goer A. et al. evaluated the results of fosfomycin susceptibility obtained using the E-test vs. the ADM for *E. coli* and *Staphylococcus* strains. The CA was 97% and 91%, respectively, i.e., above the threshold (as in our own research), while the EA was at a much higher level of 98% and 94%, respectively [34]. The VME rate for *E. coli* was unsatisfactory (12.5%), and for *S. aureus* it was perfect (0%) [34].

Karlowsky J.A. et al. used the E-test and the ADM to evaluate *Enterobacterales* other than *E. coli, P. aeruginosa*, and *S. aureus* [35]. They showed a CA, EA, and VME rate for overall *Enterobacterales* at 88.4%, 70.4%, and 32.1%, respectively; for *S. aureus* it was at 98.7%, 84.1%, and 0%, respectively; and for *P. aeruginosa* only the EA was calculated (77.6%) [35]. Their results are comparable to ours, although their VME rate for *Klebsiella* spp. was even higher.

Smith E.C. et al., in turn, evaluated the E-test for *P. aeruginosa* and showed a CA between the E-test and the ADM at a low level of 69.7% (taking into account a breakpoint of ≤128 mg/L), an EA of 81%, and a VME rate of 13.8% [8]. Peradotto M et al. also studied strains of *P. aeruginosa* (*n* = 150) and found an E-test CA of 92.7%, an EA of 74.7%, and a VME of 16.7%, with a BP of ≤128 mg/L [36].

Data on the evaluation of the susceptibility of multi-resistant strains to IV fosfomycin are also available in publications. Van Mens S.P. et al. compared the results of the E-test and the ADM in ESBL-producing *Enterobacteriaceae* strains. These authors obtained 89% CA, 57% EA, and 4% VME, with the highest agreement for *E. coli* [37]. This is one of the few reports showing a low VME rate, although it is still above the required threshold of 3%.

Camarlinghi J. et al. assessed the value of the E-test in fosfomycin susceptibility testing of KPC *Klebsiella* spp. Comparing the E-test with the ADM, they showed a CA, an EA, and a VME rate of 65.4%, 69.2%, and 78.8%, respectively [38]. However, in their studies, these authors compared the E-test to the ADM, taking into account a narrowed range of concentrations <16 mg/L through 32, 64, and 128 mg/L to >128 mg/L, and therefore their obtained EA value should be interpreted with caution. Perdigao-Neto L.V. et al. have tested carbapenem-resistant strains of Gram-negative bacilli, including *Klebsiella* spp. (100% KPC+) and *Enterobacter* spp., and *Pseudomonas* spp. The CA between the E-test and the ADM for these three genera was 52%, 40%, and 53%, respectively. The VME rate, in turn, was 50% for *Enterobacter* spp. and 0% for the others, which is a different result from that presented by other authors [39].

The results of our own research as well as those of other authors indicate that the evaluation of sensitivity to fosfomycin using the E-test does not simultaneously provide a satisfactory level of CA and VME. It can be noticed only for *S. aureus*, while for other bacteria, even if the CA is >89.9%, the VME rate is not <3%. Such unsatisfactory results are undoubtedly caused by the difficulty in reading the MIC value in the E-test, a problem that has been noticed by everyone dealing with the subject. The phenomenon of secondary growth in the zone of inhibition around the fosfomycin strip has been widely discussed. Although the rules for MIC readings provided by strip manufacturers are becoming increasingly precise, they are still not uniform. Some, such as EUCAST, recommend ignoring secondary growth, while others make this growth dependent on the type of bacteria and the number of secondary macro and microcolonies (BioMerieux, Liofilchem), and still others, such as CLSI, indicate the need to consider secondary growth in readings [1,2,22,40].

In our own research, we used an interpretation consistent with BioMerieux’s recommendations, which are described in detail in Materials and Methods. In compliance with BioMerieux instructions, macrocolonies and microcolonies were not taken into account during the reading, as shown in Figure 4. Single colonies observed in the growth inhibition zone indicate the presence of subpopulations of fosfomycin-resistant mutants. However, numerous studies have shown that the finding of such a small resistant subpopulation does not lead to an increase in resistance to fosfomycin [41,42,43]. In order to counteract this problem, intravenous fosfomycin is used in combination with other antibiotics [41,42]. Moreover, fosfomycin possesses additional properties associated with inhibition of bacterial adhesion to the epithelium and thus may prevent the spread of resistant mutants [42,43]. It has also been demonstrated that some of the mutants can inhibit bacterial adhesion by lowering the level of cAMP [43]. Though the number of colonies in the growth inhibition zone is large and the colonies fill the entire ellipse as presented in Figure 6, secondary growth is taken into account while reading, and the maximum MIC for this method was determined to be ≥1024 mg/L [22].

However, if the reading was to be performed in accordance with Liofilchem instructions, colonies filling the entire ellipse would have to be ignored, and the determined MIC value would be much lower [40]. Some authors note an improvement in categorical agreement when the reading is performed without micro- and macrocolonies, but in our study, such an omission of colonies within the ellipse would lead to even more VMEs [33,35,37]. Perhaps the discrepancies in the obtained results compared to the reference method are due to the diverse inoculum of bacteria used in different methods. In the ADM, a lower inoculum of 1×10^4^ CFU per spot is required, while in the gradient method it is 1–2 × 10^8^ CFU/mL. Ballestero-Tellez M. et al. found that as inoculum density increases, the number of resistant subpopulations increases, which is more likely to manifest itself as secondary growth [9]. It therefore appears that, since there is no consensus on the principle of strip reading, the gradient method is currently not suitable for determining susceptibility to fosfomycin. The determination of resistance to fosfomycin by the E-test seems to be more reliable because MEs occur less frequently than VMEs. Regrettably, the increasing number of strains resistant to fosfomycin will probably result in a growing incidence of such errors. Thus, even though the performance of the fosfomycin sensitivity test using the gradient diffusion method is simple, the reading of the result is not. Because the presence of secondary growth is observed in *E. coli* only occasionally, the use of gradient diffusion methods (not only the gradient strip method but also the disc diffusion method) for this type of bacteria raises the fewest doubts [44]. Unfortunately, in the case of other *Enterobacterales*, secondary growth that makes MIC reading difficult is common in gradient diffusion methods and results from the natural presence of the chromosomal *fosA* gene in most Gram-negative bacilli [42,44]. In our own research, we have abandoned using the E-test for MIC assessments for the *Proteus* genus altogether due to the swarming growth and the lack of certainty as to what value should be regarded as MIC. It is also noted that errors, including major and very major ones, may be caused by the MIC deviating from the proper one by only one two-fold dilution. In such situations, we are dealing with an essential agreement with a simultaneous lack of a categorical agreement. The worst of the errors is VME, when a resistant strain is falsely identified as susceptible to the antibiotic. In our own studies, 18 of 46 VME were associated with an E-test MIC result that was one dilution lower than the one determined by the reference method. This problem was also pointed out by Goer A. et al. in their publication devoted to the evaluation of susceptibility to fosfomycin and, in a broader context, concerning the precision of MIC reading in general, also by Doer G.V. and Brecher S.M. [34,45].

Another method that is being tested for evaluating fosfomycin susceptibility is the automated method based on microdilutions of the antibiotic in broth, such as the BD Phoenix or BioMerieux VITEK. These methods have not been approved by the FDA at all for fosfomycin susceptibility testing in the U.S. They are also not recommended in Europe, although panels with fosfomycin are available in this part of the world and can be used in laboratories [8]. The lack of recommendation for methods based on broth microdilutions (BMD) is justified, among others, by studies by Fusch P.C. et al. These authors have demonstrated on reference strains the lack of reproducibility of the obtained results of fosfomycin susceptibility tests [7]. Nevertheless, other researchers have made efforts to evaluate the reliability of this method for determining fosfomycin susceptibility. We have included the BD Phoenix system in our own research.

The panels that are an integral part of this system contain fosfomycin at concentrations ranging from 16–64 mg/L or 16–128 mg/L. Therefore, they enable susceptibility determination for *P. aeruginosa* according to EUCAST v.12. However, it is worth noting that the maximum concentration of fosfomycin >128 mg/L available in the panel is too low in the context of the new criteria for interpreting the results proposed by EUCAST in 2023 [1]. The current cut-off point for wild strains (ECOFF) of *P. aeruginosa* and fosfomycin is 256 mg/L [1]. The evaluation of susceptibility to fosfomycin using the BD Phoenix showed a similar percentage of susceptibility among the overall tested strains as the ADM (82.7%-677/919 vs. 83.2%-681/819). However, in the case of *Enterobacterales*, CPE, *Klebsiella* spp., and *Enterobacter* spp., the Phoenix was more likely to indicate susceptibility to fosfomycin than the ADM, by approximately 5%, 15%, 6%, and 13%, respectively. However, in the case of *Pseudomonas* spp., the automated system indicated resistance of the strains more often than it actually occurred (by about 15%). Using the BD Phoenix, the required CA threshold of >89.9% was obtained for *E. coli* (98.89%-179/181), the *Staphylococcus* genus (94.41%-152/161), including *S. aureus* (100%-104/104), and for *Proteus* spp. (95.2%-39/41). For the remaining groups of tested bacteria, the CA for the BD Phoenix was lower than the required value and also lower than the value obtained by the E-test. A VME rate below 3% was determined only for *S. aureus* (0%) and for MBL-producing *Pseudomonas* spp., similar to the E-test method. For the remaining groups of bacteria, very high VME rates were obtained, including higher than in the E-test method for overall strains, *Enterobacterales*, *Enterobacter* spp., *Staphylococcus* spp., and CNS. On the other hand, the Phoenix yielded a significantly lower VME rate compared to the E-test in the study of *E. coli* (by more than 9%) and ESBL-producing *Enterobacterales* (by 3%). However, this does not change the fact that testing using the Phoenix system contributed to high VME rates ranging from 9.09% to 53.83%. Overall, more VMEs were found in the BD Phoenix than in the E-test (50 vs. 46).

Similar to our research, a high CA for the Phoenix was also determined for *E. coli* (99.5%) by the previously cited van den Bijllaard et al. study. This team not only evaluated the Phoenix but also BioMerieux’s VITEK 2, obtaining a CA of 99% for *E. coli*. Unlike in our own research, van den Bijllaard et al. also obtained a high CA for *Klebsiella* spp. with automated systems. The values ranged from 93% to 94.5%, depending on the type of system. Unfortunately, the VME rate was not acceptable for either *E. coli* or *Klebsiella*, with 12–12.5% for the Phoenix and 18.8–16% for the VITEK 2, respectively [33]. Camarlinghi J. et al. evaluated the susceptibility to fosfomycin using the VITEK system, apart from the previously discussed E-test. They determined a low CA of only 52.6% for KPC-producing *Klebsiella* spp. and a VME rate of 9.1% [38].

## 5. Conclusions

Both the E-test and the Phoenix have turned out to be reliable in assessing IV fosfomycin susceptibility only for *S. aureus* (with a CA > 89.9% and a VME < 3%). For the remaining tested groups of strains and genera, the simultaneous high CA rate and low VME rate required by ISO were not achieved. Both methods fared particularly badly in detecting strains resistant to IV fosfomycin, which was reflected in a very high VME rate (up to 54% in the Phoenix for *Enterobacter* spp.). A higher VME rate and lower CA were shown for the Phoenix method compared to the E-test.

## Figures and Tables

**Table 1:** Criteria for clinical interpretation of MIC values according to EUCAST v.12 [23].

Group of Bacterial Strains	MIC Breakpoints (mg/L)	
	≤S	>R
*Enterobacterales*	32	32
*P. aeruginosa*	128	128
*Staphylococcus* spp.	32	32

**Table 2:** Susceptibility to fosfomycin obtained by the ADM, the E-test, and the Phoenix.

Bacterial Strains	*N*	Susceptibility to IV Fosfomycin					
		ADM		E-Test		Phoenix	
		*n*	%	*n*	%	*n*	%
Overall (except *Proteus)*	819	681	83.20	690	84.24	677	82.70
*Enterobacterales* (except *Proteus*)	505	391	77.42	421	83.36	415	82.17
ESBL-producing *Enterobacterales*	139	105	75.53	117	84.17	107	77.00
CPE-producing *Enterobacterales*	60	28	46.70	41	68.40	37	61.70
*Klebsiella* spp.	250	164	66.00	192	76.80	179	71.60
*E. coli*	181	170	93.92	168	92.80	170	93.92
*Enterobacter* spp.	47	34	72.30	37	78.70	40	85.10
*Proteus* spp.	41	34	82.90	No assessment		36	87.80
*Pseudomonas* spp.	153	141	90.80	123	80.40	115	75.20
MBL-producing *Pseudomonas*	21	18	85.70	15	71.40	15	71.40
*Staphylococcus* spp.	161	146	90.70	146	90.70	147	91.3
*Staphylococcus aureus*	104	103	99.00	103	99.00	103	99.00
*Staphylococcus* CNS	57	43	75.40	43	75.40	44	77.20

CPE—carbapenemase-producing *Enterobacterales.*

**Table 3:** Categorical agreement and error rates of the ADM compared to those of the E-test and the Phoenix for fosfomycin for individual species of *Enterobacterales* and *Staphylococcus* spp.

	E-Test	Phoenix
*Klebsiella* spp. *n* = 250		
CA	84.00% (210/250)	79.92% (199/250)
ME	3.66% (6/164)	10.43% (17/164)
VME	39.53% (34/86)	38.37% (33/86)
*E. coli n*=181		
CA	96.68% (175/181)	98.89% (179/181)
ME	2.35% (4/170)	0.59% (1/170)
VME	18.18% (2/11)	9.09% (1/11)
*Enterobacter* spp. *n* = 47		
CA	89.36% (42/47)	82.97% (39/47)
ME	2.94% (1/34)	2.94% (1/34)
VME	30.77% (4/13)	53.83% (7/13)
*Proteus* spp. *n* = 41		
CA	No assessment	95.12% (39/41)
ME	No assessment	0% (0/34)
VME	No assessment	28.57% (2/7)
*S. aureus n* = 104		
CA	100% (104/104)	100% (104/104)
ME	0% (0/103)	0% (0/103)
VME	0% (0/1)	0% (0/1)
CNS *n* = 57		
CA	89.47% (51/57)	84.21% (48/57)
ME	6.98% (3/43)	9.3% (4/43)
VME	21.43% (3/14)	35.71% (5/14)

**Table 4:** Agreement and differences (%) in MIC values obtained by the E-test compared to the ADM.

Bacterial Strains	Essential Agreement (EA) (%)	Same (%)	±1 Two-Fold Dilution (%)	At Least ±2 Two-Fold Dilutions (%)
Overall	75	33	42	25
*Enterobacterales*	75	30	45	25
ESBL-producing *Enterobacterales*	73	25	48	27
Carbapenemase-producing *Enterobacterales*	62	22	40	38
*Klebsiella* spp.	71	24	46	29
*E. coli*	78	35	44	22
*Enterobacter* spp.	79	32	47	21
*Pseudomonas* spp.	76	30	46	24
MBL-producing *Pseudomonas*	81	48	33	19
*S. aureus*	70	45	25	30
CNS	84	47	37	16

## Data Availability

Derived data supporting the findings of this study are available from the corresponding author Beata Kowalska-Krochmal on request.
